# COVID-19: A Possible Contribution of the MAPK Pathway

**DOI:** 10.3390/biomedicines11051459

**Published:** 2023-05-16

**Authors:** Jessica Cusato, Alessandra Manca, Alice Palermiti, Jacopo Mula, Martina Costanzo, Miriam Antonucci, Mattia Trunfio, Silvia Corcione, Francesco Chiara, Elisa Delia De Vivo, Alice Ianniello, Micol Ferrara, Giovanni Di Perri, Francesco Giuseppe De Rosa, Antonio D’Avolio, Andrea Calcagno

**Affiliations:** 1 Laboratory of Clinical Pharmacology and Pharmacogenetics, Department of Medical Sciences, Amedeo di Savoia Hospital, University of Turin, 10149 Turin, Italy; 2 ASL Città di Torino, Amedeo di Savoia Hospital, 10149 Turin, Italy; 3 Unit of Infectious Diseases, Department of Medical Sciences, Amedeo di Savoia Hospital, University of Turin, 10149 Turin, Italy; 4 Unit of Infectious Diseases, Department of Medical Sciences, City of Health and Life Sciences, University of Turin, 10126 Turin, Italy; 5 Laboratory of Clinical Pharmacology S. Luigi A.O.U., Department of Clinical and Biological Sciences, University of Turin, 10043 Turin, Italy

**Keywords:** SARS-CoV-2, molecular pathway, PBMCs, inflammation mechanism, oxygenation

## Abstract

Background: COVID-19 is characterized by an uncontrolled inflammatory response with high pro-inflammatory cytokine production through the activation of intracellular pathways, such as mitogen-activated protein kinase (MAPK). Viruses are able to exploit the MAPK pathway to their advantage; this pathway relevance to severe COVID-19 is poorly described. The aim of this study was to quantify biomarkers involved in the MAPK pathway and to clarify its possible role in affecting some COVID-19-related clinical features. Methods: H-RAS, C-RAF, MAPK1, MAPK2, and ERK were quantified through ELISA, and genetic polymorphisms were evaluated through real-time PCR. Results: We prospectively recruited 201 individuals (158 positive and 43 negative for SARS-CoV-2): 35 were male, and their median age was 65 years. MAPK-related biomarker levels were increased in SARS-CoV-2-positive participants (*n* = 89) compared to negative ones (*n* = 29). Dyspnea was reported by 48%; this symptom was associated with PBMC C-RAF levels in positive participants (*p* = 0.022) and type of ventilation (*p* = 0.031). The highest degree of ventilation was used by 8% for invasive ventilation and 41% for continuous positive airway pressure (CPAP). Conclusions: This is the first study that showed a possible contribution of MAPK-related biomarkers in affecting COVID-19 clinical features, and this may be relevant for identifying COVID-19 positive participants at risk of serious complications.

## 1. Introduction

Coronavirus disease 19 (COVID-19) is the disease caused by SARS-CoV-2 that has infected 761 million people in the last three years and that has been associated with the death of 6.887 million people [1].

Several articles showed that SARS-CoV-2 could be passed through droplets and fomites. Particularly, one of the most-used ways of diffusion for SARS-CoV-2 is person-to-person; in fact, it could be transmitted inside the household, hospital, community, and other gatherings of people. It is known that the infection could be transmitted by symptomatic and asymptomatic subjects, but SARS-CoV-2 could also be transmitted via indirect contact. Consequently, virus-containing droplets are present on hands, and people can infect other people by touching their mouth, nose, and eyes [1]. Furthermore, SARS-CoV-2 has the potential to spread through the intestinal tract. Several studies demonstrated that SARS-CoV-2 could replicate effectively in human intestinal organoids and intestinal epithelium. SARS-CoV-2 can also infect the intestinal cells of bats [1].

The clinical symptoms differ with age and gender. The severity seems to be milder in patients under 60 years old, whereas older patients have a greater possibility of respiratory failure and a longer course of the disease. The patients aged ≥65 years old are at higher risk of mortality, especially patients with acute respiratory distress syndrome (ARDS) and co-morbidities. Old males with co morbidities are more likely to be affected by SARS-CoV-2 severity [1,2].

The most common clinical symptoms of COVID-19 are basically fever and dry cough. The majority of the patients showed bilateral pneumonia, leucopenia, and lymphopenia. COVID-19 is classified into three levels according to the severity of the disease: mild, severe, and critical pathology. Asymptomatic infection cases were also suggested. Typically, the majority of patients only show mild symptoms and recover. Other than respiratory illness, COVID-19 disease seems to be associated with myocardial injury, arrhythmic complications, and neurological complications, for example myalgia, dizziness, intracranial hemorrhage, hypogeusia, headache, impaired consciousness, and hyposmia. Focusing on the gastrointestinal tract, liver injury, hypercoagulability, and thrombosis have also been observed. Patients showing severe COVID-19-related symptoms can rapidly develop ARDS, sepsis, metabolic acidosis, coagulation problems, and multiple organ functional failure. In addition, SARS-Cov-2 RNA presence in blood could lead to multiple organ failure and acute cardiac injury. Some patients were admitted to the intensive care unit (ICU) due to the complications caused by COVID-19. Currently, with the advent of new therapies and with a better knowledge of the etiopathogenesis, most patients have a good prognosis [1].

It was shown that, in severe cases, a “cytokine storm” may cause interstitial pneumonia, respiratory failure, including ARDS, organ failure, and death [2]. Altered acquired immune and uncontrolled innate inflammatory responses to SARS-CoV-2 can cause these cytokine storms [3]. Specifically, this virus is able to activate pathogenic type 1 (Th1) helper T cells in order to produce cytokines favoring inflammation processes, for example, interleukin (IL)-6 and the granulocyte-macrophage colony-stimulating factor (GM-CSF). GM-CSF leads CD14+ CD16+ inflammatory monocytes to secrete an increased level of IL-6, tumor necrosis factor alpha (TNF-α), and other types of cytokines [3].

The increased pro-inflammatory cytokine production through the activation of intracellular pathways, such as mitogen-activated protein kinase (MAPK), damages airway epithelial cells, resulting in decreased ventilation, acute lung injury, and ARDS [4].

ARDS seems to start with the infection of ciliated cells in the upper airways; then, the virus spreads down to the alveoli, probably due to reduced immunoresponse (type I and type III interferon). The infection of alveolar type II cells or inflammation, leading to endothelial activation, could damage alveolar cells. The subendothelial extracellular matrix is exposed, activating platelets and the coagulation cascade, which leads to fibrin deposition. Simultaneously, immune cells, as neutrophils, are captivated, promoting inflammation and coagulation, leading to micro-thrombi formation. Platelets could lead to thrombocytopenia; macrophages could adopt a pro-inflammatory pro-fibrotic phenotypes, and they can go into phyroptosis when infected. CD16+ T-cells can promote microvascular endothelial cell injury and the release of chemokines. Finally, armed natural killer cells can express high levels of cytotoxic proteins. This situation could lead to highly inflamed and flooded lung tissue, impairing oxygen exchange and hypoxemia (ARDS) [4].

Indeed, the spike protein promotes an angiotensin II type 1 receptor (AT1) mediated signaling cascade, inducing the transcriptional regulatory molecule nuclear factor kappa-light-chain-enhancer of activated B cells (NF-κB) and activator protein 1 (AP-1)/proto- oncogene c-Fos via MAPK activation, increasing IL-6 release [5].

Similarly, TNFα-activated MAPKs cause increased TNFα expression [6]. Consequently, MAPKs function both upstream and downstream of TNFα signalling [6]. Another molecule stimulating the activation of the MAPKs signal is the vascular endothelial grow factor (VEGF). In endothelial cells, through its tyrosine kinases receptor FLT-1, VEGF activates H-RAS which forms a complex with the terminal amino portion of C-RAF [7].

Viruses can exploit MAPK pathway to their advantage; for example herpes simplex type-1 virus induces the activation of the RAF/MEK/ERK pathway for cytoskeleton rearrangement during entry [8]. Moreover, human JC polyomavirus 2, also known as John Cunningham virus, needs ERK activation for gene transcription and Ebola virus glycoprotein- requires ERK in order to induce cytotoxicity. Furthermore, hepatitis C virus depends on RAF/MEK/ERK-mediated upregulation of cytosolic phospholipase A2 for efficient particle synthesis. As a last example, flaviviruses, including the dengue virus, depend on RAF/MEK/ERK intracellular cascade for replication [8]. It is important to highlight when MEK1/2 inhibitors are used, human immunodeficiency virus infectivity and the influenza A virus, Borna disease virus and coxsackievirus B3 propagation appears to be inhibited [8].

Therefore, it seems that the MEK1/2 inhibitors show a wide role in the spreading of viruses of different families (such as positive-strand and negative-strand RNA viruses and DNA viruses) [8]. Consequently, further studies are needed in order to clarify this aspect of COVID-19.Since RAS/RAF/MEK/ERK signalling pathway is involved in the onset of inflammation and viral infections, in addition its role in coronavirus replication is poorly described and no data are available in the literature concerning its contribution to COVID-19, aim of this study was to quantify biomarkers involved in ERK related MAPK pathway and to clarify their possible role in affecting some COVID-19 clinical features.

## 2. Materials and Methods

### 2.1. Study Design and Participants Recruitment

This is a prospective study focusing on MAPK role in affecting COVID-19 positivity and severity. MAPK-related biomarkers were quantified in all the participants, comparing negative (N = 43) and positive subjects (N = 158). The extent to which these biomarkers increase COVID-19 severity was evaluated in hospitalized patients. These patients needed the clinical support and they had symptoms within two weeks. Severity was considered in terms of type of symptoms (fever, cough, dyspnea, diarrhea, nausea and vomiting, myalgia and asthenia) and oxygenation (no oxygenation, cannula, ventimask, non-invasive ventilation, reservoir, intubation). Negative participants were considered people who were negative for SARS-CoV-2 detection and without diagnosed pathologies, aged within 18–70 years.

Participants were enrolled from April to December 2020, during the first wave of pandemic, at two Health Centers in Turin, Italy (Amedeo di Savoia Hospital and City of Health and Science—Molinette Hospital). Inclusion criteria were being above 18 years of age and having the ability to express informed consent. Exclusion criteria was the inability to express informed consent. Biological samples (whole blood and nasopharyngeal swabs) were obtained from two groups: positive at SARS-COV-2 (within 15 days after symptoms) and negative participants after signing a written informed consent. The study started after obtaining the Ethics approval by the Ethics Committee (Comitato Etico Interaziendale A.O.U. Città della Salute e della Scienza di Torino, protocol no. 00171/2020).

We collected a test tube with ethylendiamino tetracetyc acid (EDTA) for genetic analysis, a green one with litium-heparine for biomarker quantification in plasma and cell preparation tubes (CPTs) for intracellular quantifications in peripheral blood mononuclear cells (PBMCs) according to a previous validated method [9].

### 2.2. SARS-CoV-2 Positivity

The SARS-CoV-2 was obtained from nasopharyngeal and oropharyngeal swabs (300 μL) stored in the Universal Transport Medium for viruses^®^ (UTM, Copan group, Brescia, Italy). SARS-CoV-2 RNA was isolated with the Magnapure instrument (Roche, Monza, Italy), in a final elution volume of 50 μL. RNA (5 μL) was analyzed with the 2019 Novel Coronavirus Real-Time Multiplex PCR (RT-PCR) kit (Liferiver Bio-Tech, San Diego, CA, USA), a multiplex TaqMan real-time PCR targeting ORF1ab, nucleocapsid, and envelope SARS-CoV-2 genes.

### 2.3. Genetic Polymorphisms Analyses

We used the ‘QIAamp DNA mini kit’ (Qiagen, Valencia, CA, USA) for genomic DNA extraction. These kits contain columns allowing the DNA purification starting from 200 µL of blood or plasma.

Allelic discrimination was assessed through the RT-PCR (BIORAD, Milan, Italy). The following allelic variants have been analysed: *RAF* 931 T > C (rs 3729931), *ERK* 966 T > C (rs 2266966) and *MAPK* 792 G > A (rs 2283792). These genetic variants were chosen accordingly to their allelic frequency in our population (Caucasian).

### 2.4. ELISA Tests

In this study, BT LAB kits (Bioassay Technology Laboratory, Birmingham, UK) were used. In particular, the direct method was used and the antibody is found on the bottom of the various wells. In a direct ELISA, the antigen is immobilized directly on the used plate and a conjugated detection antibody binds to the target protein. Then, substrate is added, producing a signal proportional to the amount of analyte in the sample. Catalogue numbers are reported in Supplementary Table S1.

C-RAF, H-RAS, MAPK1, MAPK2 and ERK (iERK) were analysed at intracellular level (PBMCs) and VEGF, HIF (hypoxia-induced factor), TNFα, vitamin D, hepcidin, ICAM-1 (intercellular adhesion molecule-1), VCAM (vascular cell adhesion molecule), MMP9 (matrix metallopeptidase-9), IL-6 and pERK in plasma. MAPK pathway-related biomarkers were evaluated in PBMCs due to their intracellular location and activity. VEGF, HIF, TNFα, vitamin D, hepcidin, ICAM-1, VCAM, MMP9 (matrix metallopeptidase-9) and IL-6 were analyzed in plasma since they act directly in this biological matrix. Finally ERK was investigated both in PBMCs and plasma to evaluated its presence within the cells and comparing with extracellular matrix (detection due to broken and damaged cells). PBMCs were isolated from 28 mL of whole blood using BD Vacutainerw CPT™ tubes (Becton, Dickinson, Franklin Lakes, NJ, USA). Tubes were centrifuged at 800× *g* for 15 min at 20 °C and then PBMCs were washed twice in ice-cold 0.9% NaCl solution. Cell number and mean cellular volume (MCV) were obtained using an automated cell counter (Z2TM Coulter Counterw, Beckman Coulter, Brea, CA, USA). According to the kit protocol, number of cells was normalized to 1 million of cells for aliquot. Consequently, biomarkers concentration referred to 1 million of cells. The cell pellets were stored in a freezing solution (fetal bovine serum + dimethyl sulfoxide 20%) at −80° until the analyses were performed.

### 2.5. Statistical Analysis

The normality was assessed through the Shapiro-Wilk test. Categorical variables were reported as numbers and percentages. Non-normal variables were described as median and interquartile range (IQR). Differences between continuous variables and genetic groups, considering the level of statistical significance (*p*-value) < 0.05 were investigated through Kruskal-Wallis and Mann-Whitney tests. Spearman tests was used in order to evaluate correlations (SC, Spearman coefficient). Variables predictive power was analyzed through univariate and multivariate logistic regression analyses (*p*-value = *p*; *odd ratio* = OR; Interval of confidence = IC 95%).

IBM SPSS Statistics 27.0 for Windows software (Chicago, IL, USA) was used to perform the statistical analyses.

## 3. Results

### 3.1. Patient Characteristics

We recruited 201 participants: 158 (78.6%) resulted positive for SARS-CoV-2 detection, whereas 43 resulted negative. SARS-CoV-2 negative participants had a median age of 54 years (IQR 47; 66.5 years) and 18 (41.9%) subjects were male. Participants were all Caucasian. For positive participants the median age was 66 years (IQR 55; 76 years) and 107 (67.7%) participants were male: their hematochemical characteristics are summarised in Table 1.

Consequently, biomarkers were quantified in 65 PBMCs samples (36 positive and 29 negative for SARS-CoV-2) and in 149 plasma samples (135 positive and 14 negative).

### 3.2. Differences in Biomarkers Concentrations according to SARS-CoV-2 Positivity

**Figure 1:**
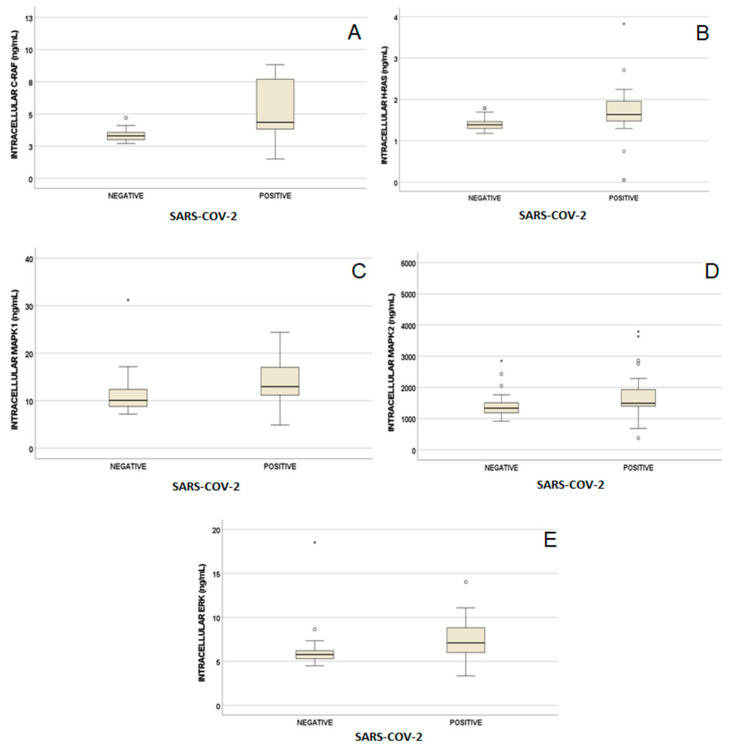
PBMCs biomarker values according to positivity to SARS-CoV-2. Intracellular ERK levels are reported for negative (left) and positive (right) participants (**A**–**E**). Circles and stars indicate “out” values (small circle) and “far out” values (star).

PBMCs biomarkers concentrations were evaluated in negative and positive participants: statistically higher levels were observed for C-RAF (*p* < 0.001, Figure 1A), H-RAS (*p* < 0.001, Figure 1B), MAPK1 (*p* = 0.001, Figure 1C), MAPK2 (*p* = 0.047, Figure 1D) and IERK (*p* < 0.001, Figure 1E).

Median concentrations were respectively for negative and positive participants 3.3 (3.0–3.6) vs.4.4 (3.8–8.3) ng/mL for C-RAF, 1.4 (10.3–1.5) vs. 1.6 (1.5–2.0) ng/mL for H-RAS, 10.1 (8.8–12.5) vs. 13.0 (11.1–17.2) ng/mL for MAPK1, 1334 (1172–1538) vs. 1492 (1393–1937) ng/mL for MAPK2 and 5.8 (5.3–6.2) vs. 7.1 (6.0–8.9) ng/mL for IERK.

Moreover, ROC was used to define possible intracellular biomarkers cut-off values predicting positivity (Table 2).

### 3.3. Biomarker Levels in COVID-19 Positive Participants

In this study, symptoms data were available for 147 SARS-CoV-2 positive patients: fever was present in 108 (73.5%) patients, cough in 69 (54.3%), dyspnea in 61 (48%), diarrhea in 21 (16.7%), nausea and vomiting in 10 (5%), myalgia in 9 (4.5%) and asthenia in 24 (20.5%).

PBMCs C-RAF levels were associated with dyspnea in COVID-19 affected participants (*p* = 0.022).

Considering the type of oxygenation (available only for 100 positive participants), the type of ventilation (comparing no oxygenation, cannula + ventimask and non-invasive ventilation (NIV) + reservoir + intubation) was associated with dyspnea (*p* = 0.033 and a difference in pERK level was highlighted between the absence of oxygen use and ventilation (*p* = 0.046).

### 3.4. Genetic Analyses

The allele frequencies of the genetic variants were showed in a Supplementary Table S2: the Hardy-Weinberg equilibrium was calculated and respected for all polymorphisms, with the exception of ERK 966 T > C.

Considering the possible impact of genetic polymorphisms, *MAPK* 792 GA/AA (*p* = 0.045) and *ERK* 966 TC/CC (*p* = 0.024) genotypes influenced H-RAS levels in PBMCs. Furthermore, MAPK 792 AA was associated with fever (*p* = 0.007), *ERK* 966 CC with nausea and vomiting (*p* = 0.013) and, finally, *RAF* 931 TT/TC and myalgia (*p* = 0.044).

**Figure 2:**
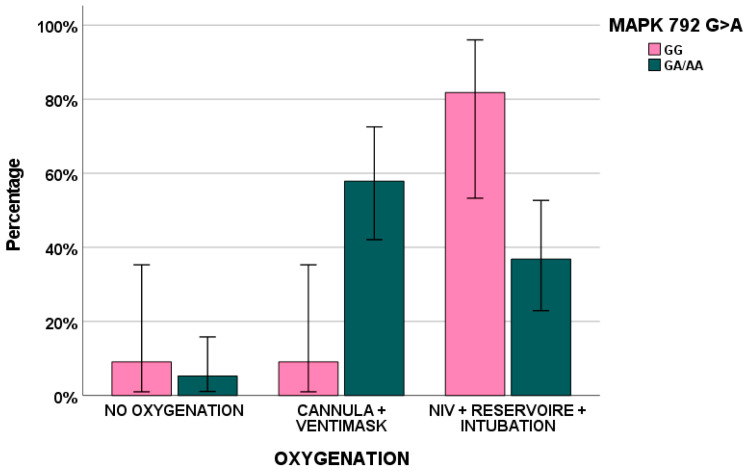
*MAPK* 792 G > A genetic variant influence on to the type of ventilation (*p* = 0.016).

In addition, *MAPK* 792 G > A was associated with the type of ventilation (*p* = 0.016), Figure 2.

### 3.5. Correlations

Finally, possible correlations among C-RAF, H-RAS, MAPK1, MAPK2, pERK and iERK vs. other COVID-19-related biomarkers (VEGF, HIF, TNFα, vitamin D, hepcidin, ICAM-1, VCAM, MMP9, IL-6) were evaluated: C-RAF with age (*p* = 0.047, S = 0.309) and plasma VEGF (*p* = 0.026, S = 0.729); MAPK1 and HIF (*p* = 0.050, S = 0.812); iERK and HIF (*p* = 0.008, S = 0.928); pERK with TNFα (*p* = 0.012, S = −0.365) and vitamin D levels at baseline (*p* = 0.024, S = −0.643). No correlation was suggested for iERK and pERK.

## 4. Discussion

It is known that the MAPK family is divided in three subfamilies: ERK, p38 and c-Jun NH2-terminal kinase (JNK). Each MAPK pathway includes three components: MAPK kinase kinase (MAP3K), MAPK kinase (MAP2K), and MAPK. MAP3Ks phosphorylate and activate MAP2Ks, which in turn phosphorylate and activate MAPKs [10]. The mechanism of this pathway started thanks to G protein-coupled receptors, which leads to the downstream molecules phosphorylation and activates the serine threonine kinase RAF (dual specificity kinase MEK and MAPK/ERK) [8].

The ERK signalling pathway influences a wide range of cellular functions, as for example cell proliferation, differentiation and survival. Since viruses are obligate intracellular parasites, these functions may play a role in virus propagation as well. After the membrane receptor stimulation, the C-RAF kinase is activated, and then it in turn phosphorylates the serine residues of MEK 1/2. After the activation, MEK 1/2 activates ERK 1/2 by phosphorylating them at the level of the serine and threonine residues. Finally, the activated ERK1/2 translocates from cytoplasm to nucleus and it is able to phosphorylate a large number of downstream substrates, such as the transcription factor c-myc, which regulates the expression of several genes [11].

Since RAF/MEK/ERK pathway seems to be involved in viral survival, Ghasemnejad- Berenji et al. in 2021 suggested this pathway as a potential therapeutic strategy for COVID-19: in fact, authors highlighted that the RAF/MEK/ERK signalling cascade is probably one of the most well-known signal transduction pathways in biology, since it is involved in a wide variety of cellular functions, such as apoptosis, cell proliferation and cell cycle arrest [8].

In the present study, 201 individuals were analysed: 158 were positive and 43 were negative for SARS-CoV-2 detection. Aim of this study was to evaluate a possible role of the ERK pathway in COVID-19. Because of this, biomarkers as H-RAS, C-RAF, MAPK1, MAPK2 and ERK were quantified in PBMCs in order to clarify their capacity in predicting SARS-CoV-2 positivity and some COVID-19 clinical features. In particular, high levels of all these five biomarkers were found in positive participants compared to negative participants, as reported in the results. As a possible explanation, these biomarkers increased levels could be probably due to ERK pathway role in affecting inflammation, which is one of the clinical characteristics of COVID-19. Furthermore, potential cut-off values associated with positivity to SARS-CoV-2 were suggested, but these data have to be confirmed in larger cohort of participants.

Aiming at strengthening this topic, correlations among some of the analysed biomarkers and plasma mediators involved in inflammation, such as VEGF and HIF, were suggested in this work. Indeed, increased C-RAF concentrations were related to higher VEGF levels and age. In addition, C-RAF was associated to dyspnea in COVID-19 affected participants. All these results could highlight the C-RAF involvement in affecting inflammation, thus dyspnea (a typical COVID-19 symptom) and age, confirming older participants have the worst clinical response.

Furthermore, MAPK1 and ERK levels were associated with HIF levels: this could emphasize the role of this intracellular cascade in affecting the severity of this pathology; consequently, higher intracellular biomarker amount was found in participants with increased HIF levels, thus with important hypoxia.

Of note, several anti-inflammatory and anti-cytokines agents have been tested in severe and at high-risk patients. Dexamethasone has been shown to reduce COVID-19 related mortality, IL-6 inhibitors to reduce risk of cardiovascular or respiratory organ support, and baricitinib to reduce time to recovery in hospitalised patients requiring oxygen support [12]. Likewise, the anti-IL-1 drug anakinra has been associated with favourable outcomes in high-risk less severe patients with COVID-19 [13].

Finally, genetics could impact on biomarker exposure and, consequently, on clinical symptoms: here, *MAPK* 792 GA/AA (*p* = 0.045) and *ERK* 966 TC/CC (*p* = 0.024) genotypes influenced H-RAS levels in PBMCs. Furthermore, *MAPK* 792 AA was associated with fever (*p* = 0.007), ERK 966 CC with nausea and vomiting (*p* = 0.013) and, finally, *RAF* 931 TT/TC and myalgia (*p* = 0.044).

*MAPK1* rs2283792 G > A is located in the intron 6 of the *MAPK1* gene: a study showed A allele increases the expression of *MAPK1* in the esophagus (muscularis) according to genotype tissue expression. We found AA genotype associated with the fever symptom: it is known that MAPK/ERK pathway is involved in the inflammation response and consequently, probably, the onset of fever [14]. Furthermore, this pathway regulates the pathogenesis of some virus infection, such as yellow fever replication and dengue [15,16]. Finally, in order to highlight our data, a study showed that Huanglian Jiedu decoction antipyretic effect is obtained by inhibiting MAPK pathway [17]: this could further support our data of AA allele associated with higher MAPK expression, considering the role of MAPK pathway in the fever onset.

*RAF1* rs3729931 SNP is located in intron 15 of *RAF1* gene. According to data from genotype tissue expression, the T allele is related to *RAF1* gene lower expression [18]. The biological significance of this SNP is currently unknown. In our study, we found *RAF* TT/TC genotype patients more predisposed to develop myalgia symptom, but this data is in contrast with what reported in literature, since these genotypes are associated to reduced RAF. Thus a better clinical outcome can be expected.

ERK2 is located on chromosome 22q11.22 and is involved in some signal transduction pathways including LH signalling during ovulation and polycystic ovary syndrome [19,20]. In addition, carriers of rs2266966 G had an association with decreased breast cancer risk in obese women [21]. A study suggests a score including the *ERK2* SNPs, rs2266966 and rs5999521, binding to many transcription factors, such as STAT2, which is involved in the defence response to virus; in fact, knockout mice for STAT 2 are more predisposed to have viral infections [22]. Moreover, our findings suggested ERK CC genotype patients are more predisposed to develop nausea and vomiting symptoms: the MAPK/ERK pathway modulates the intracellular signalling in the development of these symptoms in mice [23]. Unfortunately, the biological significance of this genetic variant is unknown.

Some of the limitations of this study are the small sample size, the cross-sectional design, the variable time from symptoms start, the differences in the controls’ characteristics and the lack of participants infected with SARS-CoV-2 omicron variants (associated with lower respiratory insufficiency despite similar lung involvement [24].

## 5. Conclusions

In conclusion, these data may help identifying patients at higher risk of severe complications thus providing a tailored anti-inflammatory treatment in addition to the routine early administration of antiviral drugs. Basically, it could be useful to perform C-RAF quantification in PBMCs in order to predict which patients could have a worst clinical outcome. Longitudinal data from larger cohorts are needed in order to verify this hypothesis.

## Figures and Tables

**Table 1:** SARS-CoV-2 positive patients hematochemical characteristics at baseline. IQR = interquartile range, CPAP: continuous positive airway pressure.

Baseline Hematochemical Values	Median [IQR]
Body mass index (Kg/m^2^)	25.7 [24.6; 29.3]
White blood cells (*n*/uL)	6180 [4590; 8790]
Lymphocytes (*n*/uL)	1040 [641; 1440]
Neutrophil (*n*/uL)	4510 [2810; 6903]
Hemoglobin (g/dL)	13.6 [12.1; 15.0]
Platelets (*n*/uL)	168,000 [12,127; 244,000]
C-reactive Protein (mg/dL)	7.1 [2.4; 12.3]
Aspartate Transaminase (U/L)	42.5 [25.5; 56]
Alanine Transaminase (U/L)	34 [22; 58]
Lactate Dehydrogenase (U/L)	329.5 [248.5; 403.5]
Creatinine (mg/dL)	0.93 [0.77; 1.20]
Procalcitonin (ng/mL)	0.1 [0.06; 0.4]
Interleukin 6 (pg/mL)	25.7 [12.7; 70.0]
Ferritin (mg/dL)	580.5 [411.5; 891.0]
D-dimer (mg/mL)	527 [260; 1399]
Fibrinogen (mg/dL)	554 [436.5; 670]
Vitamin D (ng/mL)	15.55 [10.47; 18.02]
Prothrombin Time (INR)	1.14 [1.05; 1.28]
Partial thromboplastin time (s)	29.05 [27.08; 32.0]
Troponin (ng/L)	7.5 [3; 31.25]
pH	7.47 [7.43; 7.50]
Partial pressure of oxygen in the arterial blood (mmHg)	60.75 [54.93; 68.00]
Partial pressure of carbon dioxide (mmHg)	33.00 [29.25; 36.10]
Oxygen Saturation (%)	94 [90–96]
OXYGENATION TYPE (available only for 100 participants), (*n*, %):	NO OXYGEN (10, 10%) NASAL CANNULA (23, 23%) VENTIMASK (13, 13%) RESERVOIR MASK (5, 5%) CPAP (41, 41%) INVASIVE VENTILATION (8, 8%)

**Table 2:** Receiver Operating Characteristic (ROC) curve analysis data of intracellular biomarker values predicting positivity to SARS-CoV-2. AUROC: Area Under the Roc; IC 95%: Interval of confidence at 95%.

Biomarker		*p*-Value	Value	Sensibility	Specificity	AUROC	IC 95%
C-RAF	Positivity	<0.001	3.59	83%	76%	0.840	0.735; 0.944
H-RAS	Positivity	<0.001	1.44	83%	73%	0.799	0.686; 0.912
MAPK1	Positivity	0.001	10.68	77%	55%	0.735	0.609; 0.861
MAPK2	Positivity	0.019	1337	81%	52%	0.670	0.534; 0.805
iERK	Positivity	<0.001	5.93	81%	66%	0.783	0.668; 0.898

## Data Availability

Data is contained within the article.

## Supplementary Materials

The following supporting information can be downloaded at: https://www.mdpi.com/article/10.3390/biomedicines11051459/s1, Table S1: ELISA catalogue numbers. Table S2: Allele frequencies.
