# Supplementary material for: COVID-19: A Possible Contribution of the MAPK Pathway
Source: Biomedicines. 2023 May 16;11(5):1459. doi: 10.3390/biomedicines11051459 (PMC10216575; doi:10.3390/biomedicines11051459)
Supplement: Supplementary file 1 [file biomedicines-11-01459-s001.zip › biomedicines-2338006-supplementary.docx]

Supplementary Materials

**Table S1.** Catalogue number of ELISA KITs.

| **Biomarker** | **Catalogue Number** |
| --- | --- |
| C-RAF | BTB-E2796HU |
| H-RAS | BTB-E1693HU |
| MAPK1 | BTB-E0873HU |
| MAPK2 | BTB-E2488HU |
| iERK | BTB-E0840HU |
| VEGF | BTB-E0050HU |
| HIF | BTB-E0422HU |
| TNFα | BTB-E0082HU |
| Hepcidin | BTB-E1019HU |
| ICAM-1 | BTB-E0012HU |
| VCAM | BTB-E0203HU |
| MMP9 | BTB-E0936HU |
| pERK | BTB-E0840HU |

**Table S2.** Allele frequencies**.**

| **POLYMORPHISM** | **rs** | **Allele 1** | **Heterozygous** | **Allele 2** |
| --- | --- | --- | --- | --- |
| *RAF* 931 T>C | 3729931 | 43.8 (TT) | 40.6 (TC) | 15.6 (CC) |
| *ERK* 966 T>C | 2266966 | 36.3 (TT) | 41.3 (TC) | 22.5 (CC) |
| *MAPK* 792 G>A | 2283792 | 33.8 (GG) | 39.4 (GA) | 26.9 (AA) |
